# The Expansion of a Single Bacteriophage Leads to Bacterial Disturbance in Gut and Reduction of Larval Growth in *Musca domestica*

**DOI:** 10.3389/fimmu.2022.885722

**Published:** 2022-04-06

**Authors:** Xinyu Zhang, Shumin Wang, Qian Zhang, Kexin Zhang, Wenjuan Liu, Ruiling Zhang, Zhong Zhang

**Affiliations:** ^1^ Collaborative Innovation Center for the Origin and Control of Emerging Infectious Diseases, Shandong First Medical University (Shandong Academy of Medical Sciences), Taian, China; ^2^ School of Basic Medical Science, Shandong First Medical University (Shandong Academy of Medical Sciences), Taian, China

**Keywords:** housefly larvae, intestinal bacteria, bacteriophages, microbiota-host interactions, 16S rRNA gene sequencing

## Abstract

The housefly larvae gut microbiota influences larval health and has become an important model to study the ecology and evolution of microbiota–host interactions. However, little is known about the phage community associated with the housefly larval gut, although bacteriophages are the most abundant members of the microbiota and have the potential to shape gut bacterial communities. Changes to bacteriophage composition are associated with disease, but how phages impact insect health remains unclear. We noticed that treating 1-day-old housefly larvae with ~10^7^, ~10^9^, and ~10^11^ phage particles per ml of bacteriophages led to changes in the growth and development of housefly larvae. Additionally, treating housefly larvae with bacteriophages led to bacterial composition changes in the gut. Changes in the compositions of these gut bacteria are mainly manifested in the increase in harmful bacteria, including *Pseudomonas* and *Providencia* and the decrease in beneficial bacteria, including *Enterobacter* and *Klebsiella*, after different growth and development periods. The alterations in gut microbiota further influenced the larval growth and development. Collectively, these results indicate that bacteriophages can perturb the intestinal microbiome and impact insect health.

## Introduction

The larvae of the housefly, *Musca domestica* L. (Diptera: Muscidae), are colonized with microorganisms (1) that have significant impacts on their health. The housefly larval gut provides suitable environments for microbial colonization, and bacteria in the gut potentially participate in the daily activities of housefly larvae and play important roles in many physiological functions, such as nutrition, metabolism, and immunity (2). Therefore, the larval gut microbiota of houseflies has become an important experimental model to study microbiota–host interactions. Generally, insects harbor abundant bacteria, archaea, viruses, and fungi as intestinal microbiota, forming their gut ecosystems (3). In most ecosystems, bacteriophages (phages hereafter) outnumber their bacterial prey/hosts by a factor of 10 and likely represent the most abundant foreign microorganism on the insect. Intestinal viruses, while containing members that directly infect eukaryotic cells, are largely composed of bacteriophages that target bacteria (4, 5).

Phages are predators of the bacterial world (6), and they maintain high bacterial strain-level diversity through red queen/kill-the-winner dynamics (7, 8). Because they are more genetically diverse than their bacterial prey/hosts, they are considered to play central roles in the evolution, ecology, and functioning of microbial communities (9, 10). In addition to their importance for understanding microbial community dynamics, phage–host interactions have been utilized in a variety of fields of microbiology, such as in phage therapy (11), for identifying pathogens (12), and as phage display technology (13). Despite the vital and complex contributions of phages to microbial ecology, there is a lack of knowledge about their roles in the ecology and evolution of housefly larvae-microbiota interactions.

Shifts in intestinal microbiota following phage changes can impact the health of animal hosts (14). Studies involving insect-associated phages include those from mosquitos (15), honeybees (16), black soldier flies (17), houseflies (18), etc. Phages associated with the specialized gut microbiota of housefly larvae have not been studied to date. There are no studies regarding housefly larvae-associated phages. We hypothesize that phages are likely to play an important role in modulating the bacterial community in the housefly larval gut, especially because bacterial diversity has been detected in the housefly gut microbiota (1, 2, 19, 20).

Here, we present an insect gut model to study the effects of the expansion of intestinal bacteriophages on the health of the host insect and microbiota–host interactions. We sought to isolate phages that target the intestinal strains *Enterobacter hormaechei* from housefly larvae intestines. In this study, a model intestinal phage in domestic flies was established through phage feeding experiments and amplification. We further analyzed whether the invasion of bacteriophages changed the composition of the host intestinal microbes (through 16S rRNA gene analysis) and microbiota-host interactions (by feeding bacteria to disorder the gut microbiota). The results of this study provide valuable insights into how changes in the abundance of a single phage play a role in changes in the interactions of the intestinal flora and health of insects.

## Results

### Isolation of Housefly Larval-Associated Bacteriophages of *Enterobacter hormaechei*

**Figure 1:**
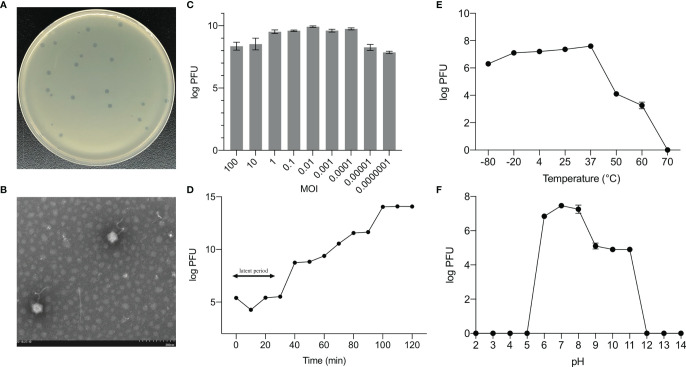
Biological properties of *E. hormaechei* phage Phc. **(A)** Morphology of bacteriophage plaques in nutrient agar medium. The plaques of Phc are medium in size and transparent. **(B)** Electron micrograph of negatively stained, purified bacteriophages used in this study. The Phc was a *Caudovirales* with uncontracted tails. **(C)** The MOI of phage Phc. *E. hormaechei* strain was infected with phage phc at various MOI (0.000001–100) and incubated at 37 C for 5 h. **(D)** One-step growth curve of phage Phc. Phage Phc has a short latent period and large burst size against *E. hormaechei*. **(E) **The thermal stability of phage Phc. Phage Phc (~10^8^ PFU/mL) was incubated at various temperatures ranging from -80 to 70°C for 1 h. **(F)** The pH stability of phage Phc. Phage Phc is stable over a relatively narrow range of pH values, and it (∼10^8^ PFU/mL) was incubated at different pH values ranging from 5 to 12 at 37°C for 1 h. Values are means ± standard deviations from triplicates of each treatment.

We identified and sequenced an *E. hormaechei*-specific bacteriophage, Phc, that was easily propagated and purified from housefly larval intestines. Phage Phc has a good lysing effect on *E. hormaechei* (**Figure 1A**) and demonstrated a narrow intraspecies host range and could not infect any of the other 8 strains isolated from housefly larval intestines, including *Pseudomonas aeruginosa*, *Klebsiella pneumoniae*, *Acinetobacter bereziniae*, *Providencia stuartii*, *Lactococcus lactis*, *Lysinibacillus fusiformis*, *Providencia vermicola* and *Bacillus safensis* (**Suplementary Figure 1**). As observed by transmission electron microscopy (**Figure 1B**), the head of the phage, which belongs to the *Drexlerviridae* family, has an icosahedral structure with an unretractable tail.

**Figure 2:**
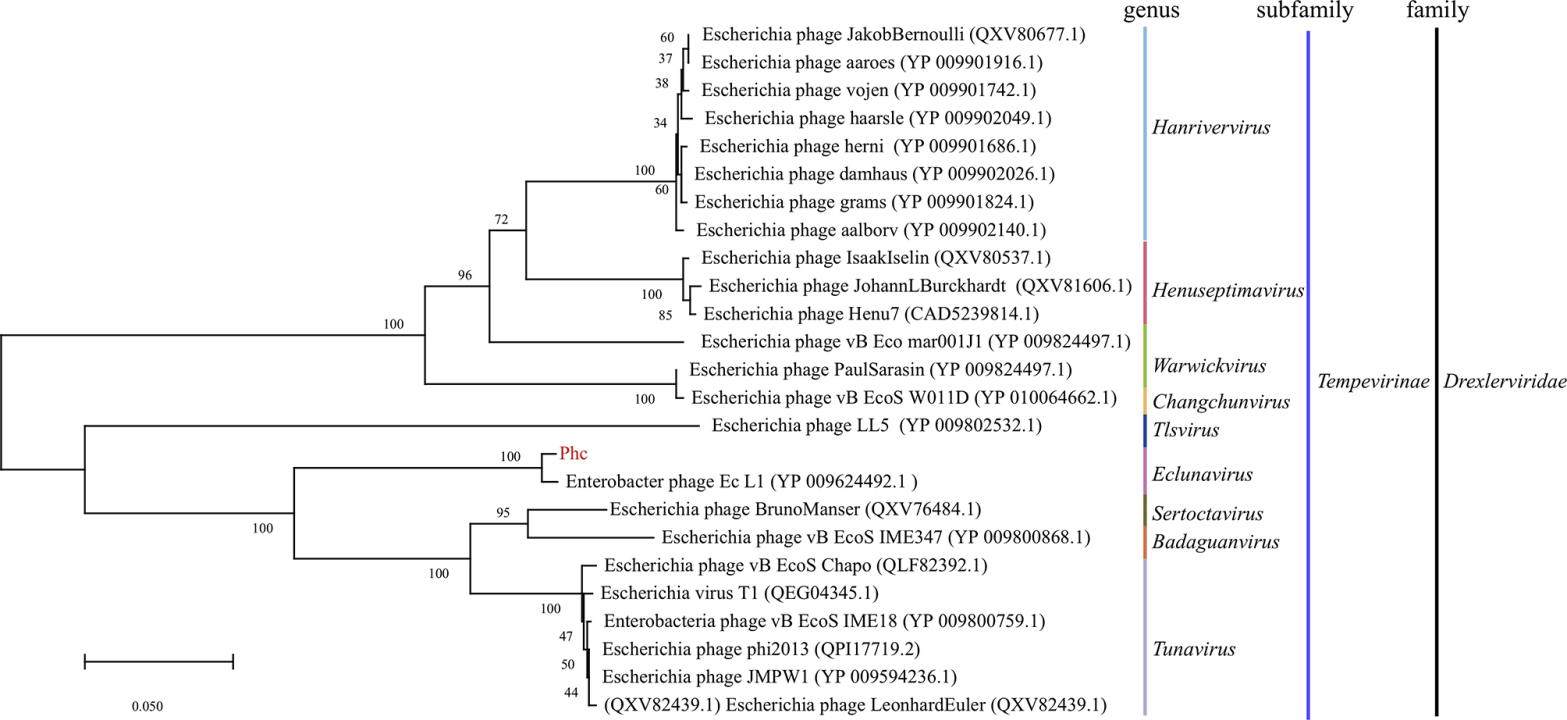
A neighbor-joining phylogenetic tree showing the relatedness of phage Phc to similar phages for which data are publicly available from the NCBI. A neighbor-joining phylogenetic tree based on terminase small subunit protein similarity between the phage used in this experiment (red color) and other similar phages for which data are publicly available from the NCBI (black color). The Phc belonged to the order *Caudovirales* and the family *Drexlerviridae* subfamily *Tempevirinae* and was highly similar to Enterobacter phage Ec_L1 (GenBank accession: NC_042122.1). The scale bar represents 0.050 amino acid substitutions per site, and values next to the nodes show bootstrap values based on 500 samples.

The bacteriophage PHc presented a latency period of 30 min, with a burst size of approximately 4.73×10^8^ particles/infected cell (**Figure 1D**). When the MOI is 10, the number of released phages is up to 8.5×10^9^ PFU/mL (**Figure 1C**). It can withstand pH5-12 environments for 1 h (**Figure 1F**). In addition, it is tolerant of temperatures from -80°C to 70°C for 1h (**Figure 1E**). In conclusion, the stability of the phage Phc is good. After genome sequencing analysis, the genome of Phc had 52,494 base pairs, with an average GC content of 36.82% (**Supplementary Figure 2**). A neighbor-joining phylogenetic tree based on terminase small subunit protein (**Figure 2**) showed that Phc (GenBank accession MZ669808) belonged to the order *Caudovirales* and family *Drexlerviridae* and was highly similar to Enterobacter phage Ec_L1 (100% similarity) (GenBank accession: NC_042122.1).

### The Effects of Bacteriophage Expansion on the Growth and Development of Housefly Larvae

**Figure 3:**
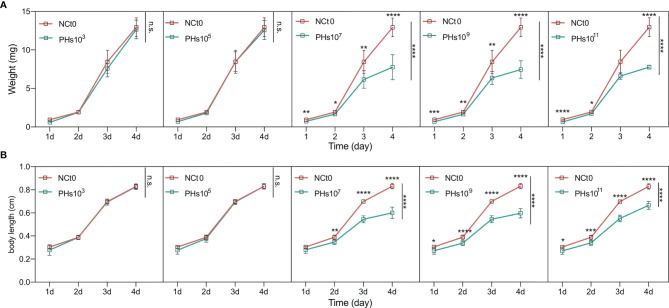
Changes in the development of housefly larvae treated with different dilutions of *E. hormaechei* ‘s phage. **(A)** The body weights of the housefly larvae changed significantly over time in the different treatments **(B)** The body lengths of the housefly larvae changed significantly over time in the different treatments. NCt0, PHs10^3^, PHs10^5^, PHs10^7^, PHs10^9^ and PHs10^11^ represent housefly larval samples treated with sterile water and sterile water containing 10^3^,10^5^,10^7^,10^9^ and 10^11^ PFU/mL phage. Data are shown as the means ± SEM. Repeated measures ANOVA was followed by Sidak correction for multiple comparisons. *P < 0.05, **P < 0.01, ***P < 0.001, ****P < 0.0001. n.s., no significance.

To amplify the intestinal phages of housefly larvae, ~10^3^, ~10^5^, ~10^7^, ~10^9^ and ~10^11^PFU/mL of the *Enterobacter hormaechei*-specific bacteriophage Phc was added to the housefly larval diet, and the body weights and lengths of the housefly larvae fed different diets were analyzed. The growth and development of the housefly larvae treated with ~10^7^, ~10^9^ and ~10^11^ PFU/mL phage were negatively affected, not ~10^3^ and ~10^5^ PFU/mL. Specifically, on the second day, the housefly larval growth began to slow, and the body lengths and weights of the larvae were lower than those of the control group (**Figures 3A, B**). These results demonstrate that compared with the control group, treatment with bacteriophages isolated from the housefly larval tract can cause bacteriophages to expand in the intestines and threaten the health of housefly larvae.

### The Effects of Increased Bacteriophage Abundance on the Housefly Larval Intestinal Microbiota

**Figure 4:**
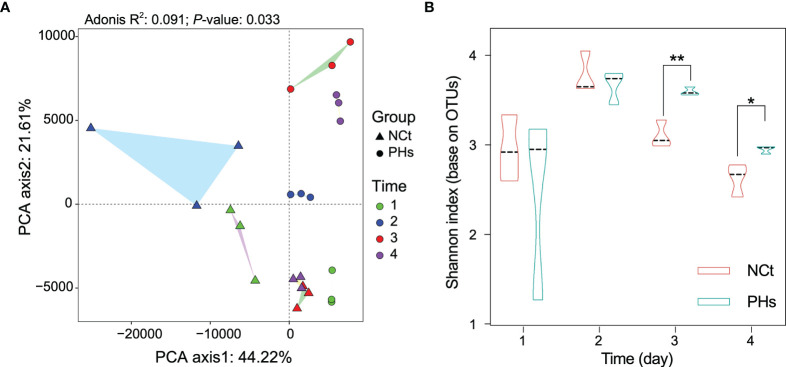
Changes in the composition and diversity of the intestinal bacterial community in different treatments. **(A)** Principal component analysis (PCA) distinguishing the dissimilarity of the OTU profiles among all the gut bacteria of housefly larvae samples. Repeated measures PERMANOVA was followed by Adonis’s correction for multiple comparisons. **(B)** Dynamics of the Shannon index of intestinal bacteria in the two groups of housefly larvae. Data are shown as the mean ± SEM. The t-test is used for statistical data. *P < 0.05, **P < 0.01.

We analyzed the intestinal specimens of housefly larvae in the phage feeding group PHs and the normal feeding group NCt from day 1 to day 4, resulting in 12 samples (NCt1, 2, 3, and 4 and PHs1, 2, 3, and 4). To examine the effects of mass amplification of phage Phc on bacterial community composition, 16S rRNA genes of housefly larval intestinal bacterial were sequenced (BioProject ID: PRJNA749627), yielding a total of 1,166,060 high-quality bacterial sequences with sequence numbers ranging from 42,148 to 57,332 per sample; these sequences were normalized and clustered into 6358 OTUs at a 97% similarity level among all the samples (**Table S2**). The presence of phages influenced the composition of the intestinal microbiome (R^2 =^ 0.091, *P*-value= 0.033, **Figure 4A** and **Table S3**). Principal component analysis (PCA) (**Figure 4A**) showed that each sample was clustered with itself; the first axis of the PCA explained 44.22% of the total variation in the bacterial community, and the second axis of the PCA explained an additional 21.61%. However, the presence of phages prevented the PHs group and the NCt group from merging. Moreover, Shannon indices suggested that the presence of bacteriophages change the bacterial diversity in the PHs group on the 3^rd^ (*P* < 0.05) and the 4^th^ (*P* < 0.01) days (**Figure 4B**). Therefore, the disturbance of the intestinal bacteria caused by bacteriophage treatment became obvious after one day.

**Figure 5:**
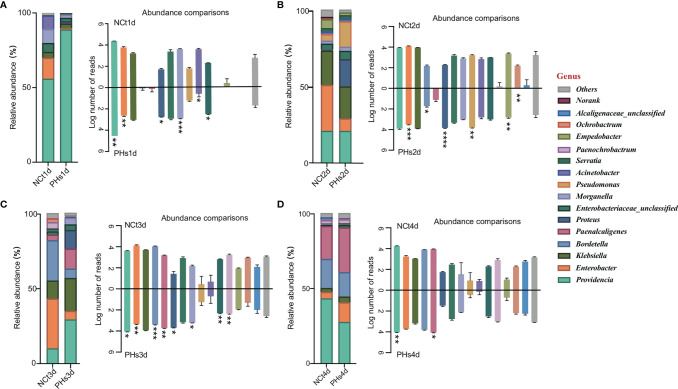
Relative abundances of the 16 most abundant genera of intestinal bacteria in phage-infected housefly larvae and the control group. Changes in the relative abundances of 16 most abundant genera in different treatments occurred on the 1^st^ **(A)**, 2^nd^ **(B)**, 3^rd^ **(C)** and 4^th^ **(D)** days and the key on the left show significant changes for each genus. Data are shown as the means ± SEM. Repeated measures ANOVA was followed by Sidak correction for multiple comparisons. *P < 0.05, **P < 0.01, ***P < 0.001, ****P < 0.0001.

Here, we selected the 16 most abundant genera for analysis. At the genus level (**Figure 5**), *Providencia* was the most abundant in housefly larvae regardless of health status after 1 day of treatment (1d hereafter) (**Figure 5A**), although its relative abundance was slightly higher in the PHs group than in the NCt group. The PHs group (1d) had more *Providencia* (88.81%) (*P*< 0.01), whereas the NCt group had slightly greater proportions of *Klebsiella* (3.91%), *Morganella* (9.30%) (*P*< 0.001) and *Acinetobacter* (8.85%) (*P*< 0.05) and notably greater proportions of *Enterobacter* (14.08%) (*P*< 0.01). For the PHs group, after 2 days of treatment (2d hereafter) (**Figure 5B**), we observed a greater proportion of *Pseudomonas* (increasing from 4.11% to 16.44%) (*P*< 0.01) as well as an increase in *Proteus* (17.68%) (*P*< 0.0001); the samples in the NCt group (2d) carried more *Enterobacter* (30.15%) (*P*< 0.001) than their phage amplification counterparts. *Providencia* was the most abundant genus in the PHs group after 3 days of treatment (3d hereafter) (**Figure 5C**), totaling 29.53% (*P*< 0.05) of the reads, followed by *Klebsiella* and *Proteus* at 22.00% and 12.44% (*P*< 0.05), respectively. The samples in the NCt group (3d) hosted more *Enterobacter* (33.27%) ((*P*< 0.01) and *Bordetella* (27.07%) (*P*< 0.001) than those in the PHs group. On the fourth day (**Figure 5D**), the healthy group (NCt) and the phage amplification treatment group (PHs) had similar genus compositions. Moreover, we observed that the proportion of *Enterobacter* increased from 4.35% to 13.13% in the PHs group. In general, the number of reads assigned to *Enterobacter* was greater in the NCt group than in the PHs group, whereas the bacterial community was dominated by different genera in the PHs group.

### The Effects of Microbiota Disturbance on the Growth and Development of Housefly Larvae

**Figure 6:**
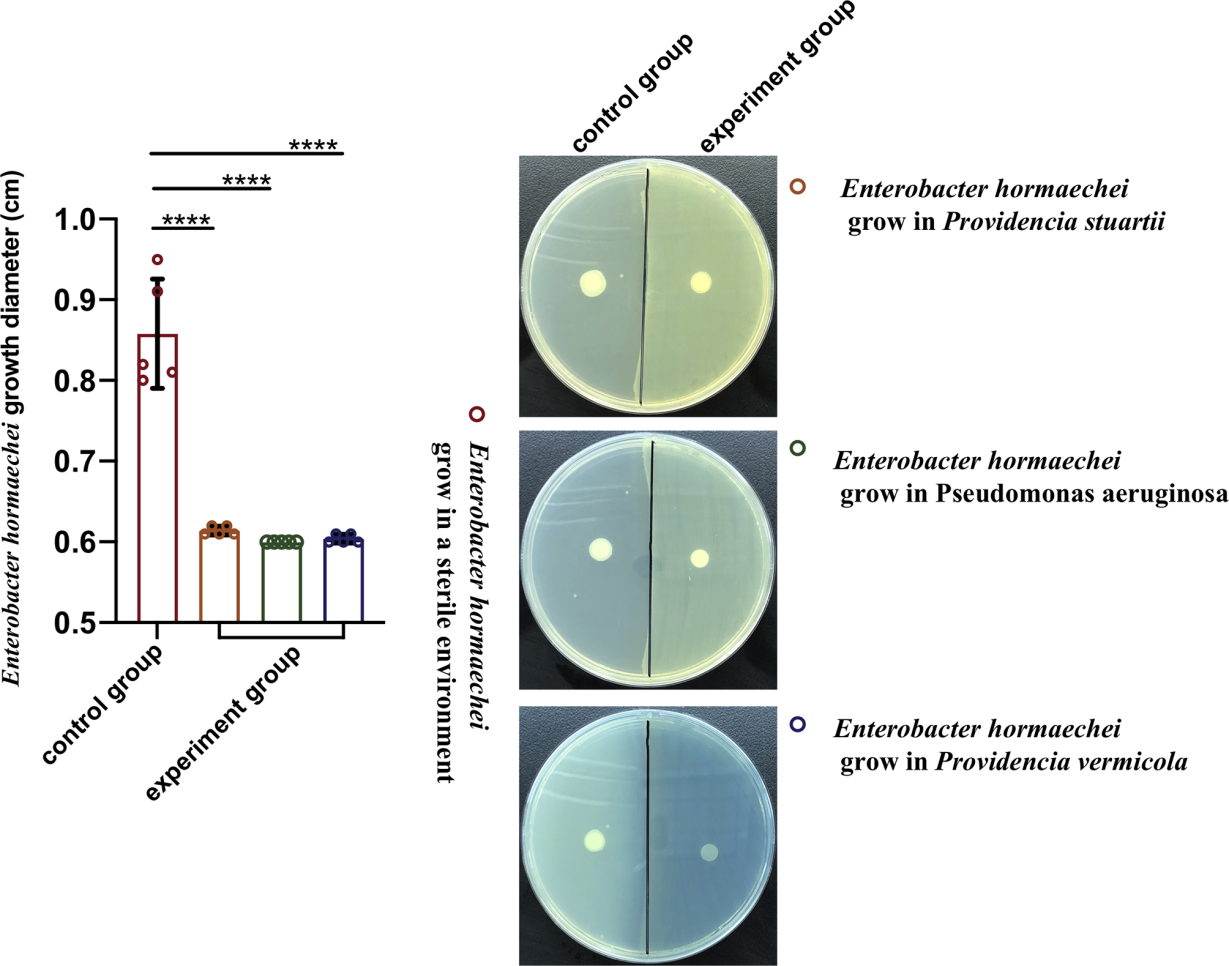
Antagonism experiment of *Enterobacter hormaechei* and other cultivable bacteria in housefly larval intestines. Data are shown as the means ± SEM. A t-test was used for the statistical analysis. ****P < 0.0001.

**Figure 7:**
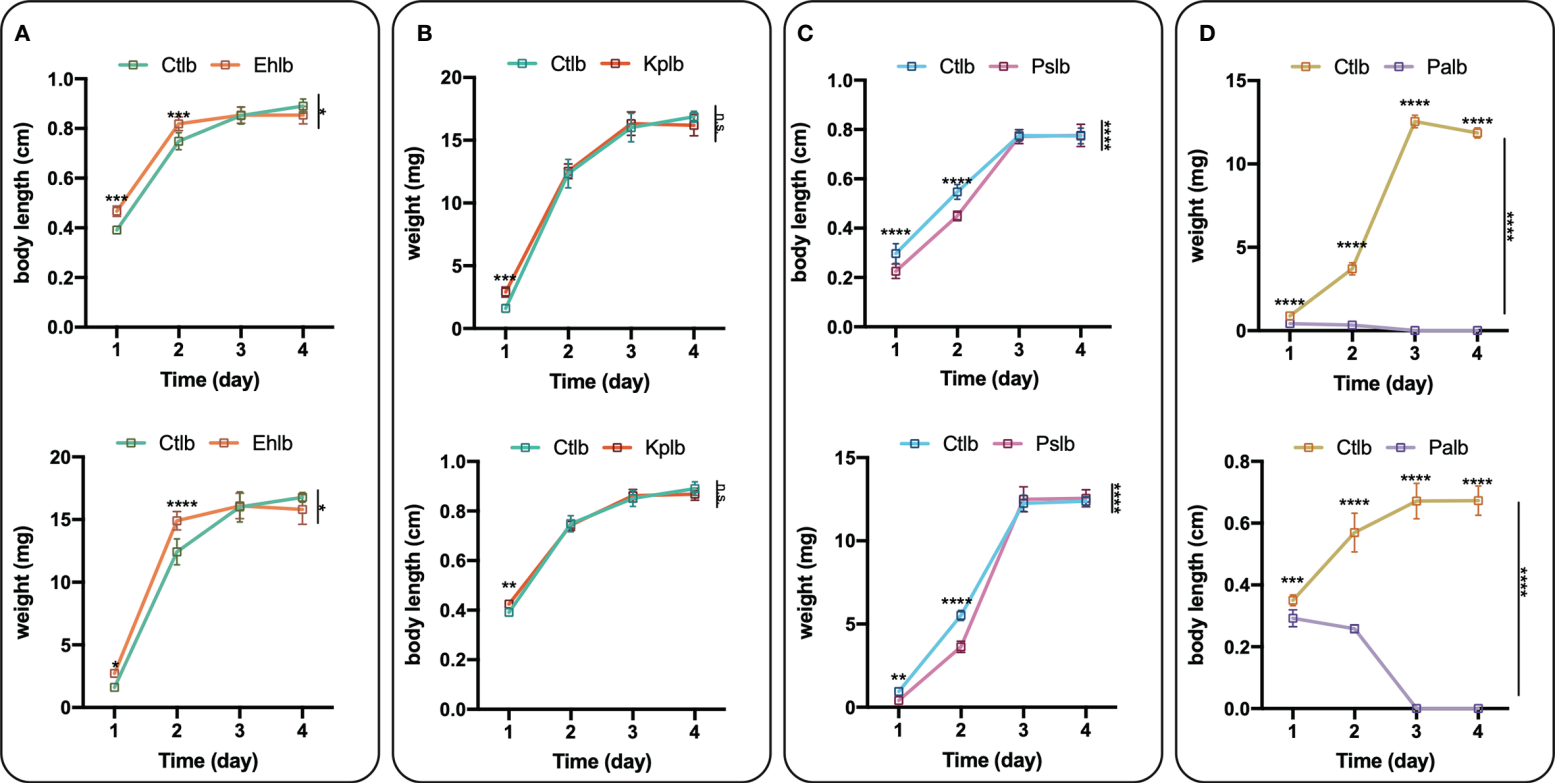
Effects of cultivable bacteria in housefly larval intestines on larval development. Ehlb **(A)**, Kplb **(B)**, Pslb **(C)** and Palb **(D)** refer to housefly larval samples fed diets with *E. hormaechei* stock solution*, K. pneumoniae* stock solution, *P. stuartii* stock solution and *P. aeruginosa* stock solution, respectively. The data are represented as the means ± SEM. Each treatment included 12 biological replicates. Repeated measures ANOVA was followed by Sidak correction for multiple comparisons. *P < 0.05, **P < 0.01, ***P < 0.001, ****P < 0.0001. n.s., no significance.

Compared with the healthy group, the increase in bacteriophage abundance caused disorders of intestinal bacteria in the treatment group. As described above, we found that the genus-level composition was quite different between groups, with varying abundances of *Enterobacter*, *Providencia*, *Pseudomonas*, and *Klebsiella*, for example. In particular, the reduction in *Enterobacter* caused by the increased abundance of phages led to changes in the abundances of other bacteria. To verify whether these bacteria were in competition with *Enterobacter*, we used a short-term *in vitro* bacterial culture experiment. We conducted an antagonism assay (**Figure 6**) with the cultivable bacterial strains *P. aeruginosa*, *P. stuartii*, *P. vermicola* and *E. hormaechei* isolated from the intestines of housefly larvae. We observed that *P.stuartii* and *P. vermicola* have an inhibitory effect on the growth of *E. hormaechei*. We speculate that the changes in the proportions of these bacteria are another key factor affecting the health of the housefly larvae. Next, we fed housefly larvae the cultivable bacterial strains *E. hormaechei*, *K. pneumoniae*, *P. stuartii* and *P. aeruginosa* (OD600 = 2.0) isolated from the intestines of housefly larvae. As shown in (**Figures 7A–D** **)**, *E. hormaechei* and *K. pneumoniae* promoted the growth of housefly larvae, whereas *P. stuartii* and *P. aeruginosa* both inhibited the growth and development of housefly larvae to varying degrees; in particular, *P. aeruginosa* had lethal effects on the larvae. Together, these results indicate that increased abundance of intestinal phages leads to a lack of beneficial bacteria and an increase in harmful bacteria that together have a negative impact on the health of housefly larvae.

### Intestinal Bacterial Symbiosis and Interaction Network

**Figure 8:**
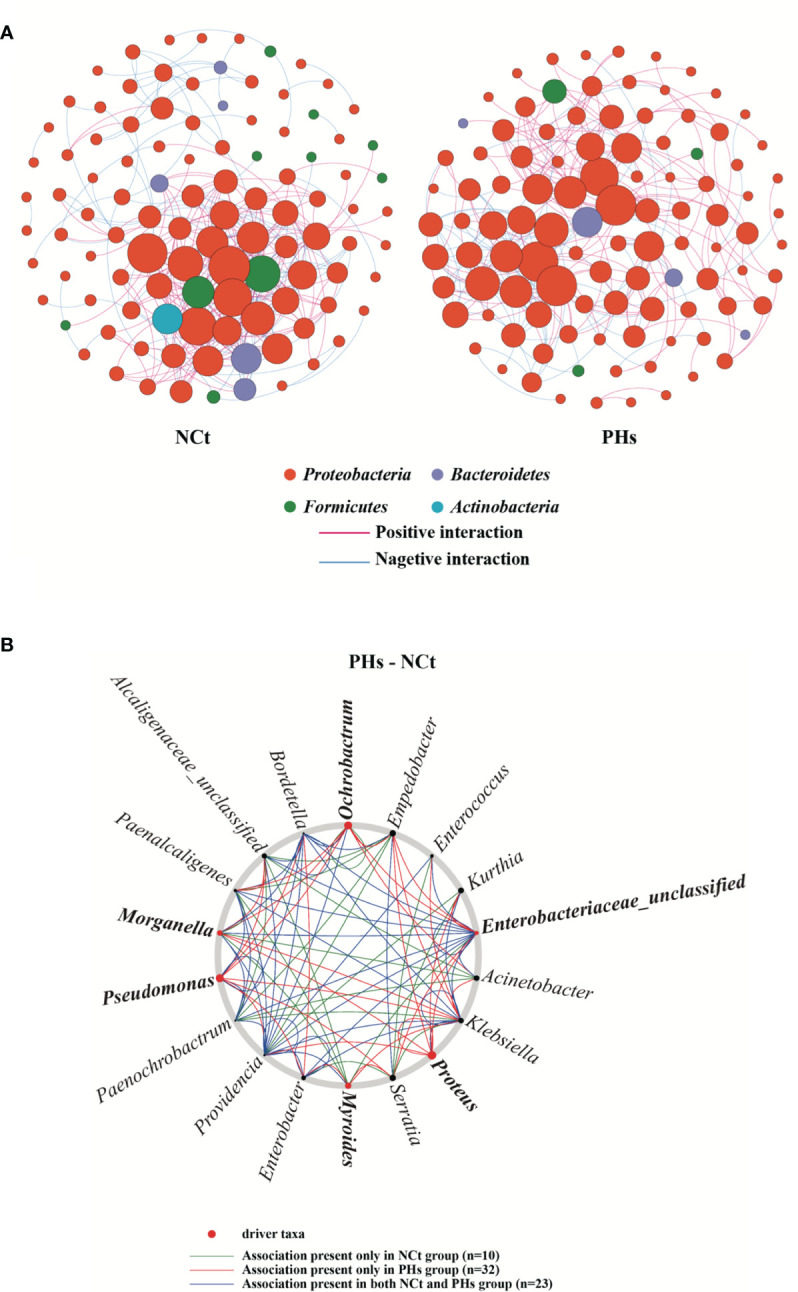
Intestinal bacterial co-occurrence microbiome networks of the NCt and PHs groups. **(A)** Effects of single phages on bacterial co-occurrence networks. Bacterial co-occurrence networks associated with single-phage experiments. Each node represents a bacterial OTU, and each edge represents a negative (displayed in blue) or positive correlation (displayed in red). The node colors represent taxon classifications at the phylum level. **(B)** Based on a NetShift analysis of the NCt and PHs groups, the potential driver taxa are essential for observing the bacterial changes in correlated network co-occurrences after phage treatment and are marked PHs-NCt. Each node size is proportional to its NESH (neighbor shift) score (a score identifying the importance of a given microbial taxon in the association network), and the nodes colored red are important driver taxa. As a result, the large red nodes represent driver taxa that are particularly important during phage infection. The line colors indicate node (taxa) connections as follows: the association appears in only the case groups (red edge), the association appears in only the control groups (green edge), and the association appears in both the case groups and the control groups (blue edge).

In this study, to analyze the interaction between bacterial communities in more detail, we built and analyzed a co-occurrence network based on OTUs. The bacterial community networks of the NCt group and the PHs group showed different symbiotic patterns. Here, we used the network topology parameter node and edge number and betweenness to evaluate the complexity of the intestinal microbial networks. The higher the number of nodes and edges was, the smaller the betweenness, and the higher the network complexity. The NCt group has many nodes and links and a small betweenness, so the network interaction of this group was more complex than that of the PHs group. Moreover, the average path length of the PHs group was greater than that of the NCt group, and there were more barriers to communication between bacterial groups in the PHs network (**Figure 8A** and **Table S4** **)**. These results indicate that the housefly larvae in the NCt group were healthier than those in the PHs group (21).

Based on NetShift analysis (22) (**Figure 8B** **)**, the associations of most taxa in the PHs group and the Ct group are completely different, and the number of important associations increases with the presence of phages. (Picture; 10 and 32 associations, respectively). In addition, separate analyses performed with NetShift revealed two potential driver groups related to changes in microbiome composition (**Figure 8B** **)**. Among these groups, we considered *Morganella*, *Proteus*, *Pseudomonas*, *Ochrobactrum*, *Myroides* and *Enterobacteriaceae_unclassified* to be the key bacterial genera in the PHs group after the increase in the abundance of the *Enterobacter hormaechei* phage Phc. These bacterial genera play an important role in changing the floral structure of the PHs group.

## Discussion

**Figure 9:**
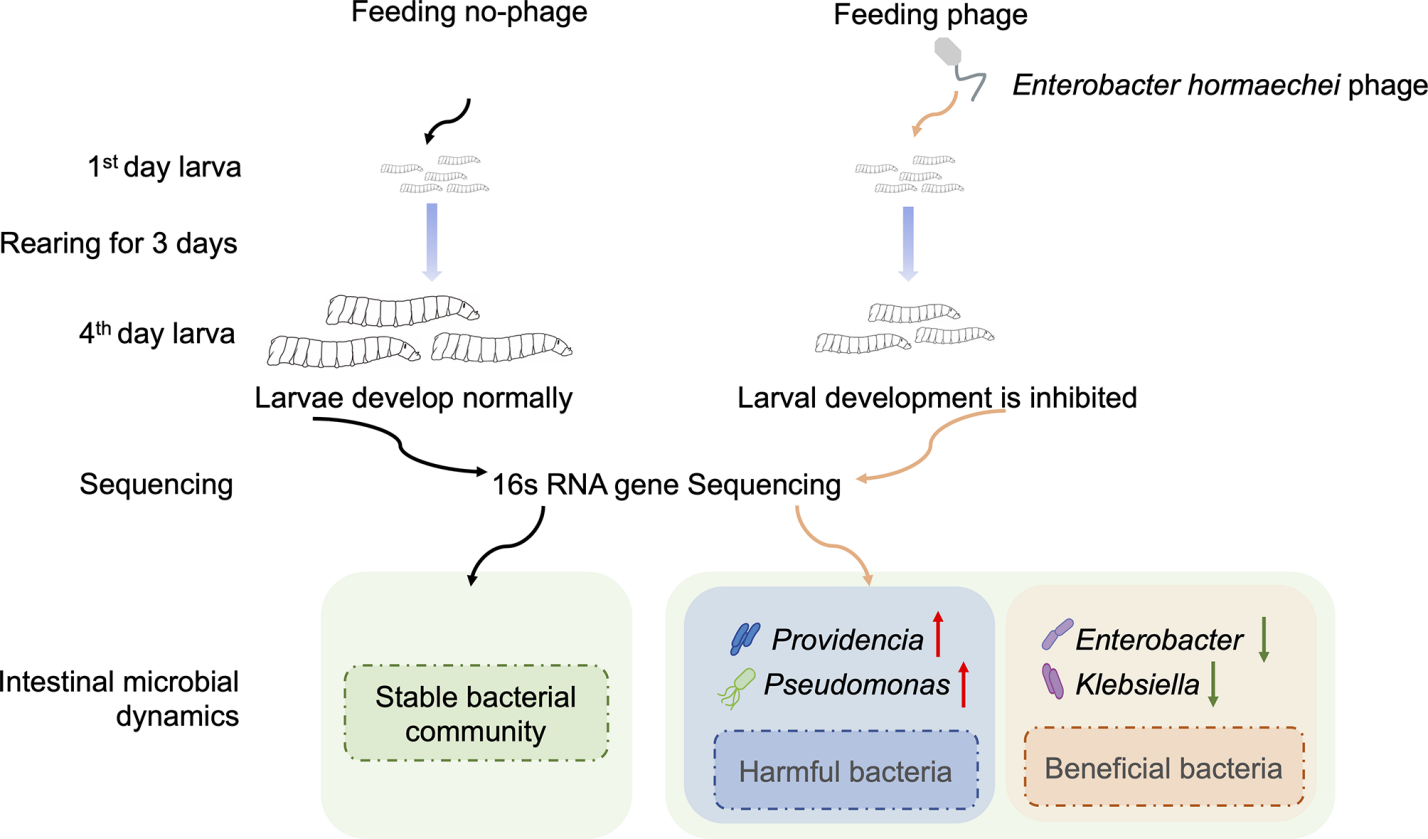
Schematic diagram of the effect of phage on housefly larvae.

The present study provides several important advances in our understanding of gut-associated phages and their specific role in the housefly larval gut microbiota (**Figure 9**). First, we show that the health of housefly larvae was negatively affected by the increased abundance of housefly larval gut bacteriophages. Second, we reveal that many invading phages can survive in the intestines for a period of time and disrupt the gut flora. Third, we demonstrate that intestinal flora disorders caused by phage amplification are an important factor affecting larvae of domestic flies, as we propagated some bacteria that are subject to significant changes *in vitro* and tested whether their increase affected the health of housefly larvae. These findings illustrate the complexity of bacteria–phage interactions in insect guts and suggest that the invasion of phages affects not only their target bacteria but also the interactions among and structure of the gut flora indirectly.

A model of intestinal phage amplification can be created by one-time administration of phages. Previous studies have shown that continuous administration of phages can significantly inhibit the growth of intestinal bacteria (23). Therefore, we consider continuous feeding of bacteria to larvae to reduce the consumption of this phage in the future. Previous studies have also shown that increasing the number of phages improved the biocontrol efficacy of combinations (24, 25). Phages in phage combinations kill pathogens through different receptors or mechanisms, which limits the evolution of bacterial resistance in phage therapy (26, 27). Only one type of phage was used in our experiment, and the bacteria may have quickly developed resistance to this phage. It is important to study the effects of multiple phage combinations on bacteria–phage interactions in insect guts in the future.

There is much research on how phages shape gut flora in mammals (28–30). However, there are few reports discussing when and how phages shape the abundances and compositions of host-associated bacteria in insect guts. Our study is the first to explore the dynamics of the gut microbiome of housefly larvae of all ages after phages invade the guts. Notably, we found that the proportions of *Enterobacter* and *Klebsiella*, etc. decreased, while the abundances of *Pseudomonas* and *Providencia*, etc. increased in the housefly larvae model of phage amplification at an age-specific stage. Because the phages we used target *E. hormaechei*, the reduction in *Enterobacter* may have been an effect of the phage targeting action. We demonstrated a narrow intraspecies host range of phage Phc, which could not infect any of the other 8 strains (*P. aeruginosa, K. pneumoniae, A. bereziniae, P. stuartii, L. lactis, L.fusiformis, P. Vermicola and B. safensis*) isolated from housefly larval intestines. Therefore, the changes in *Klebsiella, Pseudomonas*, and *Providencia* were driven indirectly by the increased phage abundance. This study shows the dynamic diversity and variation in gut bacterial communities and improves our understanding of the possible relationship between the growth and development of gut microbiomes and domestic fly larvae and stress responses to the external environment.

Changes in microbiota composition are an important factor affecting insect health (31). We propagated bacterial taxa that are subject to significant changes *in vitro* and tested whether their increased abundances in intestines affected the health of housefly larvae. Subsequently, we found that feeding housefly larvae *P. stuartii* and *P. aeruginosa* caused stunted growth, whereas feeding housefly larvae *K. pneumoniae* and *E. hormaechei* promoted their development. Our plate confrontation assays also demonstrated that *P. stuartii* and *P.s aeruginosa* significantly inhibit the growth of *E. hormaechei*, so we suspect that several bacterial groups, such as *Pseudomonas* and *Providencia*, are negatively correlated with *Enterobacter* and positively correlated with *Klebsiella*. These results also demonstrate that the increasing abundance of harmful bacteria due to the lack of beneficial bacteria is another key factor affecting the health of housefly larvae. Finally, we used a co-occurrence network to analyze potential mutual changes in gut flora. Furthermore, we were able to identify several driver taxa that could play a key role in bacterial symbiotic networks and be enriched in phage groups. NetShift analysis identified *Morganella*, *Proteus*, *Pseudomonas*, *Ochrobactrum*, *Myroides* and *Enterobacteriaceae_unclassified* as key bacterial genera after the massive amplification of phages, and these genera play an important role in changing the floral structure of the PHs group.

The use of phages in insect intestinal models for creating interference in intestinal floral composition is promising. Although the concept of using phages to control disease-causing bacteria in humans and plants is not new, phage use is still rare in insect models. We report that the interaction of phages with their bacterial hosts and their effects on bacterial composition may play an important role in the health and disease of larvae.

## Methods

### Animal and Microbial Strains

The housefly (*Musca domestica*) colony was reared and maintained in the Laboratory of Vector and Insect Diseases of Shandong First Medical University for approximately 15 years. Houseflies were reared in gauze cages (30 cm × 30 cm × 30 cm). The adult flies were provided with brown sugar and water as a diet. The diet of housefly larvae was composited with sterilized wheat bran and sterilized water (1:1), mixed to paste in proportion. The insects were kept in an artificial climate chest at 28 ± 1°C, a 45-55% relative humidity (RH) and a photoperiod of LD 16:8 h.

The bacterial strains Enterobacter hormaechei, Pseudomonas aeruginosa, Klebsiella pneumoniae, Acinetobacter bereziniae, Providencia stuartii, E. cloacae, Lactococcus lactis, Lysinibacillus fusiformis, P. vermicola and Bacillus safensis were isolated from housefly larval intestines using traditional isolation and culture methods. Strains were cultured at 37°C in LB broth (yeast extract, 5.0 g l^−1^; tryptone, 10.0 g l^−1^; NaCl, 10.0 g l^−1^) for 24 h with shaking (170 r.p.m.) before all the experiments. For long-term storage, bacterial cultures were stored in glycerol [1:1 (v:v)] at -80°C.

### Bacteriophage Isolation

We chose a lytic phage (Phc) that was isolated from housefly larval intestines as our model phage (**Table S1**). The bacterial strain *E. hormaechei* was used as an enrichment host to isolate bacteriophages isolated from housefly larval intestines.

Approximately 10 1- to 3-day-old larvae were starved for 4 h, placed in aseptic centrifugal tubes, sterilized with 75% (v/v) ethanol for 10 min, and then rinsed 3 times with sterilized water to remove bacteria from the insect surface. After rinsing, the gut contents were extracted, resuspended in 10 mL of phosphate buffered saline (PBS) and stored at -80 °C until use. For phage isolation, the gut contents were thawed, and an equal volume of LB broth was added. After incubation at room temperature for 2h the samples were centrifuged for 20 minutes at 6000 rpm. The supernatant was collected and passed through sterile syringe filters (0.22 µm, Millex-GP, Merck-Millipore, USA) to separate phages from bacteria that remained in the supernatant. Twenty milliliters of LB broth, 20 mL of gut content filtrate and a 1 mL overnight culture of *E. hormaechei* were combined and incubated with shaking at 37 °C overnight. The following day, cell debris was removed by centrifugation for 20 minutes at 6000 rpm. The supernatant was collected and filtered through a 0.22 µm filter and checked for the presence of phages by depositing 10 mL on double-layered plates containing the lawns of *E. hormaechei* cells. The top agar layer, together with phages within the clearing zones, was collected and soaked in PBS. After appropriate dilution, the suspensions were plated for plaque formation. At least three more successive single-plaque isolations were performed to obtain pure cultures. The phage solution was serially diluted, and 100 µL of each dilution was mixed with bacteria, 0.7% LB agar and 100 µL of overnight culture of the host. The suspension was carefully distributed on the top of the LB agar plates and incubated overnight at 37°C. The phage titer was calculated by multiplying the number of plaques by the dilution factor.

### Phage Biological Characteristics Assay

To determine the host range (32) of phage Phc, spot test method was used. Briefly, take 10ul 10^8^ PFU/mL phages and drop them on the bilayer agar plates containing bacterial solution(*E. hormaechei*, *P. aeruginosa*, *K. pneumoniae*, *A. bereziniae*, *P. stuartii*, *E. cloacae*, *L. lactis*, *L. fusiformis*, *P. vermicola* and *B. safensis*), and observe the growth status of bacteria on the plates after 24 h. The presence of plaques means that the bacteria are sensitive to bacteriophages, and the absence of plaques indicates that the bacteria are resistant to phages. The optimal multiplicity of infection (33) (MOI is defined as the ratio of the number of phages to the number of host bacteria) of phage Phc was determined in a coculture of *E. hormaechei*. Phage Phc and *E. hormaechei* were co-cultivated in 5mL of LB broth at different MOIs (100, 10, 0.1, 0.01, 0.001, 0.0001, 0.00001, 0.000001). The preparations were incubated with shaking at 110 rpm and 37°C. Phage titer is measured after 6 h and the largest number is the optimal number of infections. The one-step growth curve experiment (34) is as follows. 500 μL of 10^8^ PFU/mL of phage suspensions and 500 μL of optimal MOI host bacterial solution were incubated at 37°C and were taken after 5min. The mix cultures were collected and centrifuged (4°C; 10,000 rpm; 5 min) to pellet mixture of virus and bacteria. The resulting supernatant was discarded, and the pellet was resuspended in 1mL of PBS. Subsequently, the suspension resuspended in 19 ml of LB broth and incubated at 37°C. Aliquots was collected at 10min intervals for a total of 120 min and filtered through a 0.02 µm filter, and the titers determined by the double-layer agar method. Adaptability assay for phages at different pH, take 100 μL of 10^8^ PFU/mL of phage fluid into LB broth of 900ul at pH 2.0, 3.0, 4.0, 5.0, 6.0, 7.0, 8.0, 9.0, 10.0, 11.0, 12.0, 13.0 and 14.0 and incubated at 37°C. Phage titer is measured 1 h after incubation of the mixture. For temperature stability determination, take 100ul of 10^8^ PFU/ml of phage into 900ul fresh LB broth for incubation at different temperatures (-80°C, -20°C, 4°C, 25°C, 37°C, 50°C, 60°C and 70°C), and aliquots were taken after 1h of incubation. All assays were performed as described previously with some modifications and were performed in triplicate.

### Phage Electron Microscopy

Negative stain transmission electron microscopy (TEM) was used to image purified phage preparations. The purified phage (10^9^ PFU/mL) was placed on a carbon-coated copper grid, washed with deionized H2O, stained with 1% uranyl acetate for 20 seconds and subsequently air-dried. The specimen was blotted with filter paper between steps.

### Phage Genome Sequencing and Bioinformatics Analysis

Phage chromosomal DNA was isolated using the λ phage genomic DNA purification kit (ABigen) following the manufacturer’s instructions. Whole-genome sequencing was performed with an Illumina HiSeq 4000 platform. The _METAVIRAL_SPA_DES_ pipeline [DOI: 10.1093/bioinformatics/btaa490] was used to identify the phage in the sample (mainly including sequence assembly, phage sequence identification, and phage integrity identification). Gene predictions and annotations were carried out using GeneMarkS. Annotated genome map was made using the criclize package in R. Phage sequences were deposited in the NCBI and accession numbers are MZ669808 (Phc). Single Protein Based Phylogenetic Analysis: A neighbor-joining phylogenetic tree based on phage tail fiber protein similarity between the phage Phc used in this experiment and other 24 similar phages that are publicly available at NCBI (**Figure 2**). Phylogenetic trees, based on 500 bootstrap replicates, were constructed by employing Neighbor-Joining (NJ) methods using MEGA 6.0 (35).

### Phage Infection in a Housefly Larval Model

The initial phage stocks were prepared by growing phages with the stock *E. hormaechei* strain in LB medium for 24 h as described above, with the addition of centrifugation (10 min at 8,000 g) and filtration (0.22 μm) steps to isolate and purify phages from bacteria. The phage titers were adjusted to ~10^3^, ~10^5^, ~10^7^, ~10^9^ and ~10^11^ phage particles per ml, and the phage stocks were stored at 4°C. We selected 1-day-old housefly larvae hatched for a 4-day feeding experiment to test the impacts of phage expansion in the presence of a natural intestinal microbiome. The diet of the housefly larvae was composed of sterilized wheat bran and sterilized water (1:1), which were mixed to paste. The sterile water of the experimental group contained ~10^3^, ~10^5^, ~10^7^, ~10^9^ and ~10^11^ total phage particles per ml. The control treatment did not include the addition of phages. The larvae in the experimental group were treated in the same way as those in the control group for the remaining 3 days. Ten 1-day-old larvae were reared in a 5 ml perforated test tube (a total of 15 tubes were made) to ensure ventilation and placed in an incubator at a temperature of 26 ± 1°C, a relative humidity of 60% ± 10%, and an illumination ratio of 12:12 (L:D). For each group, the experiment was carried out in three perforated test tubes independently, and 4 samples were taken from each test tube every day as replications. The body lengths and weights of the housefly larvae were recorded daily. Housefly larvae that treated with sterile water and sterile water containing 10^9^ PFU/mL phage were collected from the test tubes daily until 4 days after treatment. The surfaces of the housefly larval samples were thoroughly cleaned with sterile water, and then the whole intestine was dissected under sterile conditions. The dissected intestines were washed with sterile water 3 times and then placed in sterile centrifuge tubes containing sterile normal saline, with one sample per tube, and the sample number was marked on the tube. Finally, ten intestinal samples were mixed as a sample unit. Each sample unit was a pool containing ten intestinal samples from phage-infected housefly larvae or controls. Three units of 24 repetitions were used for 16S rRNA high-throughput sequencing of intestinal bacteria.

### DNA Extraction of the Intestinal Bacteria

The intestinal samples were homogenized in a tissue lyser (Qiagen, Hilden, Germany) followed by genomic DNA isolation using the Wizard Genomic DNA Purification Kit (Promega; A1120) according to the manufacturer’s instructions, with DNA suspended in 30 μl nuclease-free water. The concentration and quality of extracted DNA were assessed using a NanoDrop 2000 spectrophotometer (Thermo Fisher Scientific, Waltham, MA, USA) and 2% agarose gel electrophoresis, respectively. Extracted DNA was stored at -20°C until further processing.

### Determining Changes in Gut Composition Using Illumina MiSeq Sequencing

The hypervariable V3-V4 region of the bacterial 16S rRNA gene was amplified with the primers 341F (5’-CCTAYGGGRBGCASCAG-3’) and 806R (5’-GGACTACNNGGGTATCTAAT-3’). Quality control of the original data was carried out using Trimmomatic v0.39 software (http://www.usadellab.org/cms/index.php?page=trimmomatic). Based on the overlap (minimum: 10 bp) between PE reads after quality control, PE reads were assembled using FLASH v1.2.11 software (FLASH: fast length adjustment of short reads to improve genome assemblies). QIIME v1.9.1 software (36) (QIIME allows analysis of high-throughput community sequencing data) was adopted for processing, and VSEARCH v2.14.1 software (VSEARCH: a versatile open-source tool for metagenomics) was used for detecting chimeric sequences. Based on a sequence similarity level of 97%, the UCLUST method in QIIME software was employed to perform OTU clustering analysis. Based on the Silva reference database (Release 138), taxonomic annotations were made for the OTUs in each sample. Sequencing data of the microbiome was deposited in the Sequence Read Archive (SRA) database and can be accessed by the BioProject accession number PRJNA749627.

### Plate Confrontation Assay Between *E. hormaechei* and Other Cultivable Bacteria

To determine the interactions between the other cultivable bacteria (*K. pneumoniae*, *P. aeruginosa*, *P. stuartii* and *P. vermicola*) and *E. hormaechei*, we conducted plate confrontation experiments on NA medium plates (peptone, 10.0 g l^−1^; agar, 20 g l^−1^; NaCl, 5.0 g l^−1^; and beef extract, 3.0 g l^−1^) in a microaerobic environment. *E. hormaechei* and other cultivable bacteria were inoculated in LB liquid medium (yeast extract, 5.0 g l^−1^; tryptone, 10.0 g l^−1^; and NaCl, 10.0 g l^−1^) and cultured at 37°C overnight (the concentration of the bacterial solution was adjusted to OD_600 =_ 1). The other cultivable bacterial cultures were inoculated with a sterile cotton swab on half of a nutrient agar plate using the spread plate method, and the opposite side of the agar plate was used as a negative control. Six-mm-diameter sterile filter papers were dipped in *E. hormaechei* bacterial liquid. After slight drying, the filter papers were placed on the two sides of the agar medium coated with other bacteria. All the plates were incubated at 37°C and placed in an anaerobic incubator for 24 hours. Finally, the bacterial growth diameter was recorded. The colony sizes of *E. hormaechei* and other cultivable bacteria were measured to evaluate their interactions. The experiments were conducted with six independent biological replications.

### Cultivated Bacteria to Feed Housefly Larvae

*E. hormaechei*, *K. pneumoniae*, *P. aeruginosa*, *P. stuartii* and *P. vermicola* were inoculated in LB liquid medium (yeast extract, 5.0 g l^−1^; tryptone, 10.0 g l^−1^; and NaCl, 10.0 g l^−1^) and cultured at 37°C overnight (OD_600 =_ 2) before the feeding experiment. The diet of one-day-old housefly larvae was composed of paste made from sterilized wheat bran and the cultured bacterial liquid described above (1:1), whereas the control group was provided the same amount of LB broth. Other than their slightly different diets, the growth conditions of the control group were the same as those of the larvae infected with the phages, as described above. The body lengths and weights of the housefly larvae were recorded daily. For each group, 6 sample repetitions were recorded per day.

### Bacterial Co-Occurrence Networks

Network analysis was conducted only between single- and three-phage treatments to specifically focus on phage effects on the microbiome. Networks were drawn using Gephi (37), and the ‘NetShift’ method was used to identify potential keystone driver taxa underlying differences in microbiomes exposed to single-phage and three-phage communities.

### Statistical Analysis

All data were analyzed using SPSS Statistics v.20 and Prism 8 (GraphPad software). The remaining statistical analyses were conducted in the R open-source software (version: V4.1.2, http://www.r-project.org/). Beta diversity indices, including the PCA were analyzed using the “vegan” package in R, using the Bray-Curtis dissimilarity. The graph constructed using the ggplot2 package. PERMANOVA analyses were performed using the Adonis function from the Vegan package.

## Data Availability Statement

The datasets presented in this study can be found in online repositories. The names of the repository/repositories and accession number(s) can be found in the article/**Supplementary Material**.

## Author Contributions

RZ and ZZ conceived and directed the project together. XZ, SW, QZ, KZ, and WL performed the experiments. XZ and SW analyzed the results and wrote the manuscript. RZ and ZZ revised the manuscript. All authors contributed to the article and approved the submitted version.

## Funding

This work was supported by the National Natural Science Foundation of China (No.81572028 and 81871686).

## Conflict of Interest

The authors declare that the research was conducted in the absence of any commercial or financial relationships that could be construed as a potential conflict of interest.

## Publisher’s Note

All claims expressed in this article are solely those of the authors and do not necessarily represent those of their affiliated organizations, or those of the publisher, the editors and the reviewers. Any product that may be evaluated in this article, or claim that may be made by its manufacturer, is not guaranteed or endorsed by the publisher.

## Supplementary Material

Supplementary Figure 1 Resistance of *E hormaechei* and other cultivable bacterial isolates in housefly larval intestines to the phages used in the experiments. The infectivity of the phage Phc against host bacteria and nonhost bacterial isolates (other cultivable bacteria) from housefly larval intestines.

Supplementary Figure 2 Annotated genome map for the phage Phc used in the experiments. In the circular genome map, the outermost black circle represents the full length of the genome, the innermost multicolored circle represents the annotated functional protein, the second outermost blue circle represents the GC content, and the third outermost purple circle represents the GC skew.
