# Supplementary figures and images for: The Expansion of a Single Bacteriophage Leads to Bacterial Disturbance in Gut and Reduction of Larval Growth in Musca domestica
Source: Front Immunol. 2022 Apr 6;13:885722. doi: 10.3389/fimmu.2022.885722 (PMC9019163; doi:10.3389/fimmu.2022.885722)

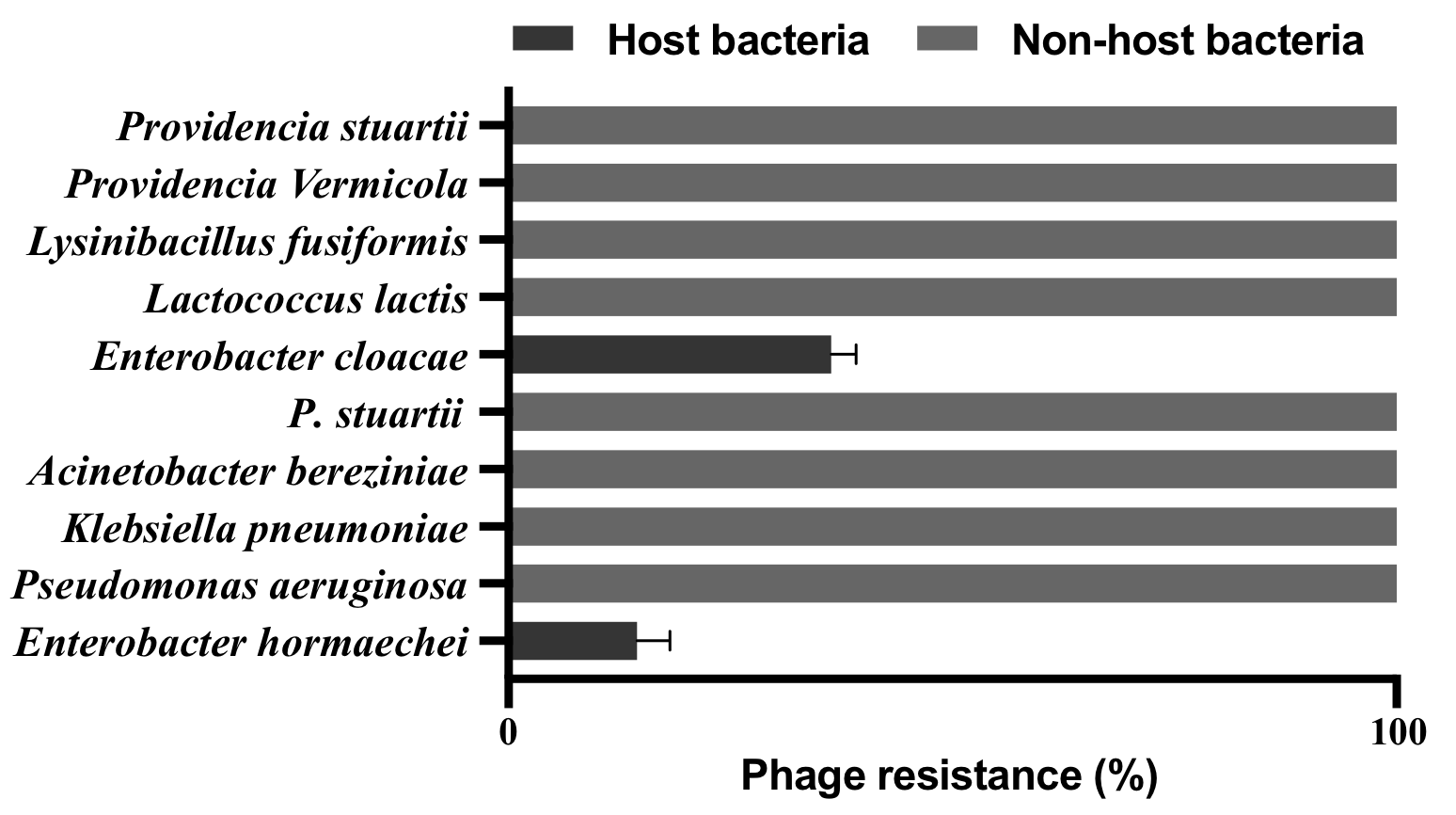

Supplement: Supplementary Figure 1 — Resistance of E hormaechei and other cultivable bacterial isolates in housefly larval intestines to the phages used in the experiments. The infectivity of the phage Phc against host bacteria and nonhost bacterial isolates (other cultivable bacteria) from housefly larval intestines. [file Image_1.tiff]

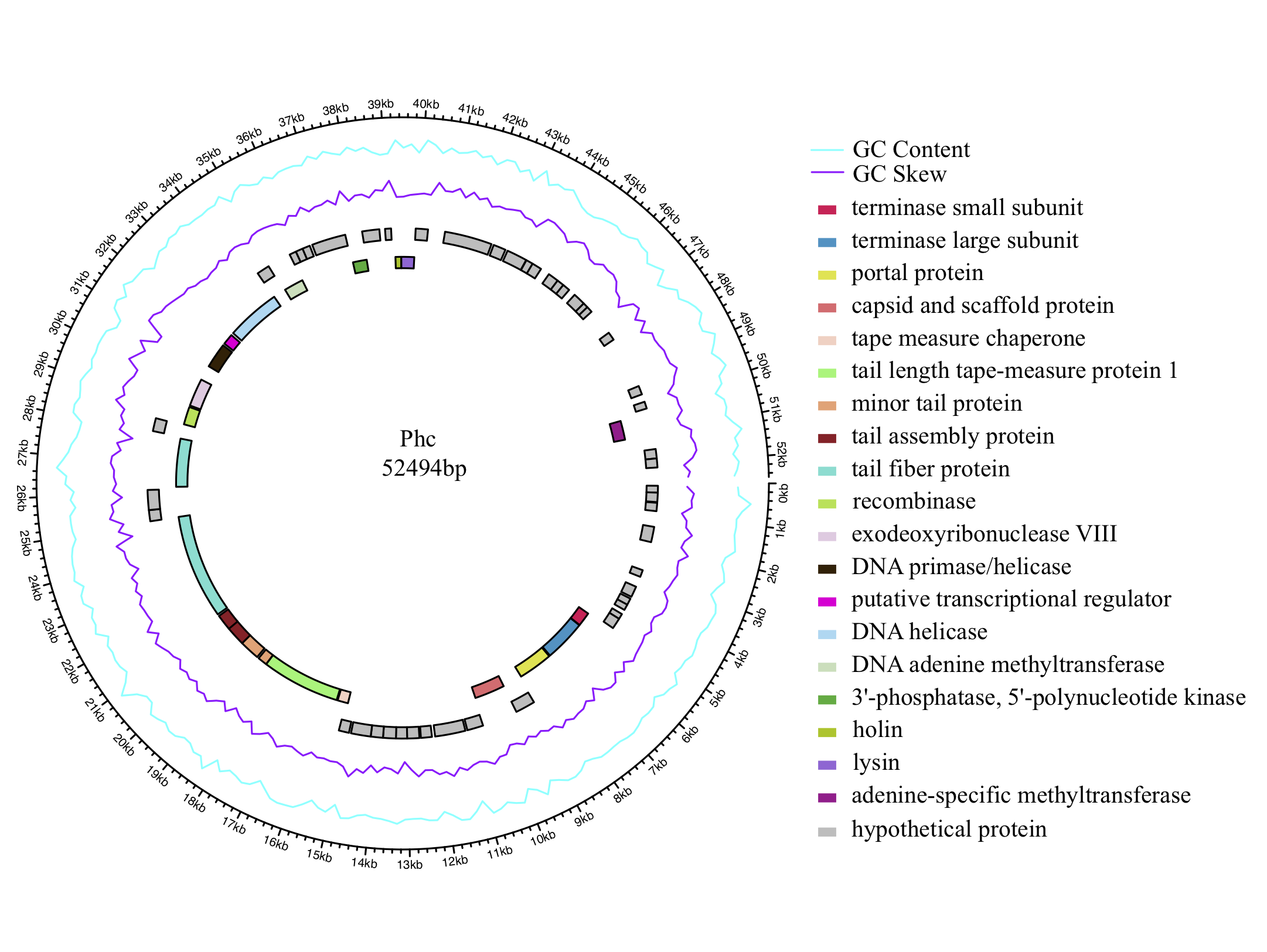

Supplement: Supplementary Figure 2 — Annotated genome map for the phage Phc used in the experiments. In the circular genome map, the outermost black circle represents the full length of the genome, the innermost multicolored circle represents the annotated functional protein, the second outermost blue circle represents the GC content, and the third outermost purple circle represents the GC skew. [file Image_2.tiff]
