# Supplementary material for: The Expansion of a Single Bacteriophage Leads to Bacterial Disturbance in Gut and Reduction of Larval Growth in Musca domestica
Source: Front Immunol. 2022 Apr 6;13:885722. doi: 10.3389/fimmu.2022.885722 (PMC9019163; doi:10.3389/fimmu.2022.885722)
Supplement: Supplementary file 3 [file Table_1.docx]

**Supplementary materials**

**Table S1** Table showing the names, locations, years of isolation and morphologies of the phage used in this study.

| **Name** | **Source and year** | **Morphology** |
| --- | --- | --- |
| Phc | Housefly larva intestine  2019 | *Caudovirales (*order*)*  *Drexlerviridae (*family*)* |
