# Supplementary material for: The Expansion of a Single Bacteriophage Leads to Bacterial Disturbance in Gut and Reduction of Larval Growth in Musca domestica
Source: Front Immunol. 2022 Apr 6;13:885722. doi: 10.3389/fimmu.2022.885722 (PMC9019163; doi:10.3389/fimmu.2022.885722)
Supplement: Supplementary file 4 [file Table_2.docx]

**Supplementary materials**

**Table S2** Information derived from the 16S rRNA gene analysis in this study. Data are expressed as the mean ± standard deviation of three replicate samples in each sampling.

| **Samples** | | **Clean**  **Reads** | **OTU number** | **Shannon** | **Simpson** | **Chao1** | **Coverage** |
| --- | --- | --- | --- | --- | --- | --- | --- |
| **NCt1** | **NCt1_1** | 49990 | 552.33±76.89 | 2.95±0.37 | 0.1285±0.0298 | 536.00±66.30 | 0.9981±0.0004 |
|  | **NCt1_2** | 53757 |  |  |  |  |  |
|  | **NCt1_3** | 54964 |  |  |  |  |  |
| **NCt2** | **NCt2_1** | 49195 | 810±289.51 | 3.78±0.24 | 0.0615±0.0123 | 815.33±286.82 | 0.999±0.0005 |
|  | **NCt2 _2** | 41253 |  |  |  |  |  |
|  | **NCt2_3** | 47977 |  |  |  |  |  |
| **NCt3** | **NCt3_1** | 41070 | 372.33±49.54 | 3.11±0.15 | 0.0615±0.0123 | 379.67±47.69 | 0.9991±0.0002 |
|  | **NCt3_2** | 40243 |  |  |  |  |  |
|  | **NCt3_3** | 43993 |  |  |  |  |  |
| **NCt4** | **NCt4_1** | 43286 | 334.67±22.48 | 2.62±0.18 | 0.1595±0.0400 | 346.67±18.61 | 0.999±0.0001 |
|  | **NCt4_2** | 40181 |  |  |  |  |  |
|  | **NCt4_3** | 44393 |  |  |  |  |  |
| **PHs1** | **PHs1_1** | 52843 | 806.67±477.01 | 2.47±1.04 | 0.3315±0.2567 | 819±488.61 | 0.9988±0.0011 |
|  | **PHs2_2** | 48585 |  |  |  |  |  |
|  | **PHs1_3** | 51592 |  |  |  |  |  |
| **PHs2** | **PHs2_1** | 45988 | 439.33±45.8 | 2.47±1.04 | 0.3315±0.2567 | 442.33±44.12 | 0.9995±0.0001 |
|  | **PHs2_2** | 43694 |  |  |  |  |  |
|  | **PHs2_3** | 40679 |  |  |  |  |  |
| **PHs3** | **PHs3_1** | 43736 | 410.67±45.17 | 3.35±0.39 | 0.0563±0.0034 | 413.67±46.11 | 0.9995±0.0001 |
|  | **PHs3_2** | 43596 |  |  |  |  |  |
|  | **PHs3_3** | 46966 |  |  |  |  |  |
| **PHs4** | **PHs4_1** | 48585 | 321.67±29.02 | 2.95±0.04 | 0.1195±0.0067 | 336.67±22.03 | 0.999±0.0002 |
|  | **PHs4_2** | 43815 |  |  |  |  |  |
|  | **PHs4_3** | 40066 |  |  |  |  |  |
