# Supplementary material for: The Expansion of a Single Bacteriophage Leads to Bacterial Disturbance in Gut and Reduction of Larval Growth in Musca domestica
Source: Front Immunol. 2022 Apr 6;13:885722. doi: 10.3389/fimmu.2022.885722 (PMC9019163; doi:10.3389/fimmu.2022.885722)
Supplement: Supplementary file 5 [file Table_3.docx]

**Supplementary materials**

**Table S3** The PCA score for each sample

| **Group** | **PCA score** | |
| --- | --- | --- |
|  | **PCA1** | **PCA2** |
| NCt1d_1 | -6220.5686 | -1301.4413 |
| NCt1d_2 | -4309.5268 | -4557.7307 |
| NCt1d_3 | -7442.7494 | -355.95064 |
| PHs1d_1 | -11721.711 | -93.935878 |
| PHs1d_2 | -25122.357 | 4536.48122 |
| PHs1d_3 | -6435.3976 | 3478.66801 |
| NCt2d_1 | 2461.25144 | -5302.5689 |
| NCt2d_2 | 993.921986 | -6220.1197 |
| NCt2d_3 | 1726.39393 | -4894.8909 |
| PHs2d_1 | 512.248048 | -4472.2427 |
| PHs2d_2 | 1563.26813 | -5010.005 |
| PHs2d_3 | 1439.29438 | -4341.3961 |
| NCt3d_1 | 5436.70473 | -3938.2575 |
| NCt3d_2 | 5363.80358 | -5833.2263 |
| NCt3d_3 | 5359.51438 | -5679.1562 |
| PHs3d_1 | 2678.21687 | 413.749492 |
| PHs3d_2 | 1503.45805 | 631.285323 |
| PHs3d_3 | 178.071283 | 580.83928 |
| NCt4d_1 | 5418.01196 | 8278.58682 |
| NCt4d_2 | 173.081612 | 6870.57135 |
| NCt4d_3 | 7740.17514 | 9681.73873 |
| PHs4d_1 | 5977.8122 | 6520.75789 |
| PHs4d_2 | 6258.8359 | 6052.24949 |
| PHs4d_3 | 6468.2462 | 4955.9942 |
