# Supplementary material for: The Expansion of a Single Bacteriophage Leads to Bacterial Disturbance in Gut and Reduction of Larval Growth in Musca domestica
Source: Front Immunol. 2022 Apr 6;13:885722. doi: 10.3389/fimmu.2022.885722 (PMC9019163; doi:10.3389/fimmu.2022.885722)
Supplement: Supplementary file 6 [file Table_4.docx]

**Supplementary materials**

**Table S4** Topological properties of bacterial co-occurrence networks associated with no-phage and single-phage treatments.

| **Group** | **Network indices** | | | | |
| --- | --- | --- | --- | --- | --- |
|  | **Network index** | **Total nodes** | **Total links** | **Maximal betweenness** | **Average clustering coefficient** |
| **NCt** | 0.860 | 105 | 296 | 1208.835 | 0.198 |
| **PHs** | 0.820 | 104 | 269 | 1776.305 | 0.234 |
